# Editorial Notes

**Published:** 1905-03

**Authors:** 


### Editorial Notes.

**University College Colston Society.**

THE sixth annual meeting of this Society was held on January 31st, 1905, in the large hall of the College, under the presidency of the Right Honourable Lewis Fry, who was supported by the vice-president (Mr. J. W. Arrowsmith), the Master of Trinity College, Cambridge (Canon H. M. Butler, D.D.), the Principal of Birmingham University (Sir Oliver Lodge, F.R.S.), the Lord Bishop of Bristol, the Lord Bishop of Hereford, and the Right Honourable Henry Hobhouse, M.P. Many speakers reverted to the long-discussed question of a University of Bristol, the chairman remarking that the existing system of education in the city could not be complete until a university linking all the existing institutions together and dominating the whole with intelligence had been established. The Bishop of Bristol remarked that it would be difficult to find two men who could better represent the old and the new in university ideals than the guests of the evening, the Master of the greatest of the colleges of the old universities and the Principal of what he might describe as the greatest of the new universities. Both of these had much to say on the subject of university extension. Sir Oliver Lodge spoke of the local university as an educational centre of influence to schools, technical institutes, and all sorts of educational organisations: it might be the educational head of a province. He thought it likely that provinces would revive in England, and that Bristol should be the capital of Wessex in a very real sense, and that its university should be the University for Wessex. He considered that a university should leaven the life of a city, and that a great city without its university was but an uncrowned queen. This was an ideal to be worked for by every lover of the higher education in the West, inasmuch as an institution which could give the stamp of accredited degrees to its alumni would raise the whole intellectual status of the city, and would in time react on the civic life as well. The Master of Trinity College, Cambridge, spoke on the great value and importance of the College, and on the necessity for union between the two great branches of learning, science and literature. He further spoke on the much-vexed question of compulsory Greek, and, although a profound lover of the Greek language and literature, he was convinced that there are a great number of boys for whom the double burden of the two most difficult languages was too great. He believed in the value even of translations of the classics, and hoped that the library of the College might be richly stored with the best translations of Latin and Greek books, so that all students might be allowed the intense enjoyment and duty of saturating themselves through these translations with the thought, the life, the religion, the very genius of these two immortal languages.

**The Prevention of Apoplexy.**

THE subject of Professor Clifford Allbutt's address to the Bristol Medico-Chirurgical Society on January 11th was one of great interest and of profound importance. The difficult task of the prevention of apoplexy was surveyed, and the whole subject of the association of arterio-sclerosis and high arterial pressure, with its measurement by the various forms of sphygmo-manometers, was reviewed. The causes of this condition, and the principles of its treatment by means of mercurials, salines, and iodides, formed the basis of an address (page 1), which we are pleased to publish *in extenso*, and which cannot fail to be instructive and profitable to our readers. The one weak point in the estimation of arterial pressure lies in the numerous fallacies which attach themselves to the clinical use of the instruments devised for the purpose, and until these can be more readily and accurately employed the physician will be likely to continue to attach more importance to the inferences to be drawn from information obtained by the fingers than to any observations recorded by the sphygmograph, arteriometer, or hæmodynamometer.

**The alleged Abuse of Hospitals.**

In a recent number (vol. xxii., p. 272) we directed attention to the subject of hospital administration, and published a paper by Miss Elizabeth Sturge on "The Misuse of Medical Charities." This perennial subject is ever cropping up anew, and now it has again come to the front by the allegations in the columns of the *Times*, made more particularly by Sir Henry Burdett, that the hospitality of the great medical charities is systematically abused. These statements have been supported by Mr. W. Roger Williams, of Clifton, who states that Bristol, with a population of 340,000, provides annually the large number of 140,000 with gratuitous medical relief. He estimates that some 40,000 persons in Bristol are annually in receipt of medical relief who cannot and ought not to plead poverty. He further states that if these undeserving crowds were eliminated the funds at present available for charitable medical purposes would be amply sufficient for all legitimate requirements. Probably there is little difference between the metropolitan and the provincial hospitals with regard to this matter. The tendency of mankind to get what they can for nothing is everywhere the same, and the difficulty in deciding who are eligible for gratuitous relief is also the same everywhere. The medical profession has been in the habit of giving the applicant the benefit of the doubt: he gets relief first, and an inquiry as to his circumstances is made a secondary matter. It is impossible to trust to appearances, as the most needy-looking is often the most undeserving, and the most tidy-looking are commonly those who are struggling the hardest. The statistics quoted are open to many fallacies, and the abuse is probably far less than it is made to appear on paper. The numbers are swelled largely by the crowd of what are called emergencies, many of these of the most trivial character, and which could be dealt with at very small cost by the local practitioners. Many other trivial cases apply with notes available for six weeks' treatment, when one visit to any medical practitioner would suffice, and this could be obtained for a very small fee. The cases which really are the most desirable are the chronic cases which require persistent and expensive treatment, and also those which require special skill or special appliances such as cannot be adequately provided by the general practitioners around. For all such a rigid wage-limit is an impossibility, and the practitioners themselves are often the greatest sinners by sending patients to the hospital for treatment when they could well afford to pay ordinary fees at home. Some abuse is inevitable, and will never be absolutely stopped, but it is clear that the conscience and the judgment of the applicants themselves are most fallacious guides.

